# Term infant formula supplemented with milk-derived oligosaccharides shifts the gut microbiota closer to that of human milk-fed infants and improves intestinal immune defense: a randomized controlled trial

**DOI:** 10.1093/ajcn/nqab336

**Published:** 2021-10-07

**Authors:** Elvira Estorninos, Rachel B Lawenko, Eisel Palestroque, Norbert Sprenger, Jalil Benyacoub, Guus A M Kortman, Jos Boekhorst, Jodi Bettler, Colin I Cercamondi, Bernard Berger

**Affiliations:** Asian Hospital and Medical Center, Muntinlupa City, Philippines; Asian Hospital and Medical Center, Muntinlupa City, Philippines; Asian Hospital and Medical Center, Muntinlupa City, Philippines; Nestlé Institute of Health Sciences, Nestlé Research, Lausanne, Switzerland; Immunology, Nestlé Research, Lausanne, Switzerland; NIZO Food Research BV, Ede, The Netherlands; NIZO Food Research BV, Ede, The Netherlands; Nestlé Product Technology Center—Nutrition, Société des Produits Nestlé S.A., Vevey, Switzerland; Nestlé Product Technology Center—Nutrition, Société des Produits Nestlé S.A., Vevey, Switzerland; Nestlé Institute of Health Sciences, Nestlé Research, Lausanne, Switzerland

**Keywords:** milk-derived oligosaccharides, infant formula, gut microbiota, bifidobacteria, opportunistic pathogenic bacteria, intestinal immune response, gut maturation

## Abstract

**Background:**

Bovine milk-derived oligosaccharides (MOS) containing primarily galacto-oligosaccharides with inherent concentrations of sialylated oligosaccharides can be added to infant formula to enhance the oligosaccharide profile.

**Objective:**

To investigate the effects of an MOS-supplemented infant formula on gut microbiota and intestinal immunity.

**Methods:**

In a double-blind, randomized, controlled trial, healthy term formula-fed infants aged 21–26 d either received an intact protein cow milk-based formula (control group, CG, *n* = 112) or the same formula containing 7.2 g MOS/L (experimental group, EG, *n* = 114) until the age of 6 mo. Exclusively human milk-fed infants (HFI, *n* = 70) from an observational study served as the reference. Fecal samples collected at baseline, and the ages of 2.5 and 4 mo were assessed for microbiota (16S ribosomal RNA-based approaches), metabolites, and biomarkers of gut health and immune response.

**Results:**

Aged 2.5 and 4 mo, redundancy analysis (*P* = 0.002) and average phylogenetic distance (*P* < 0.05) showed that the overall microbiota composition in EG was different from CG and closer to that of HFI. Similarly, EG caesarean-born infants were different from CG caesarean- or vaginally born infants and approaching HFI vaginally born infants. Relative bifidobacteria abundance was higher in EG compared with CG (*P* < 0.05) approaching HFI. At the age of 4 mo, counts of *Clostridioides difficile* and *Clostridium perfringens* were ∼90% (*P* < 0.001) and ∼65% (*P* < 0.01) lower in EG compared with CG, respectively. Geometric LS mean (95% CI) fecal secretory IgA in EG was twice that of CG [70 (57, 85) compared with 34 (28, 42) mg/g, *P* < 0.001] and closer to HFI. Fecal oral polio vaccine-specific IgA was ∼50% higher in EG compared with CG (*P* = 0.065). Compared with CG, EG and HFI had lower fecal calcium excretion (by ∼30%, *P* < 0.005) and fecal pH (*P* < 0.001), and higher lactate concentration (*P* < 0.001).

**Conclusions:**

Infant formula with MOS shifts the gut microbiota and metabolic signature closer to that of HFI, has a strong bifidogenic effect, reduces fecal pathogens, and improves the intestinal immune response.

## Introduction

Human milk is finely attuned to the needs of infants supporting optimal growth and overall development. In addition to the nutritional components, it contains important bioactive components, such as enzymes, growth factors, antimicrobial compounds, oligosaccharides, and immunological factors (1, 2). Emerging evidence suggests that the distinct array of oligosaccharides in human milk provides a variety of physiologic benefits to infants, including the establishment of a balanced gut microbiota (3, 4), prevention of pathogen adhesion to mucosal surfaces (5), modulation of the immune response (6, 7), and potential support to brain development (8). Currently, most infant formulas do not contain human milk oligosaccharides and their absence may contribute to differences in health outcomes that have been observed between human milk- and formula-fed infants (9).

Infant formula composition is developed after human milk, the gold standard, and is evolving with ongoing research on human milk composition and properties as well as technological progress. A promising novel approach to enhance the oligosaccharide profile of infant formulas is the addition of bovine milk-derived oligosaccharides (MOS). Bovine milk contains a variety of neutral and acidic oligosaccharides (10) and advances in technology now allow enriching these oligosaccharides from bovine milk whey and/or whey permeate (11). MOS are primarily composed of galacto-oligosaccharides with inherent concentrations of sialylated oligosaccharides which are structurally identical to some of the sialylated oligosaccharides in human milk (12). These sialylated oligosaccharides contribute to the structural diversity of MOS providing sialic acid and multifaceted monosaccharides linkages. Thus, the addition of MOS may functionally improve infant formulas and beneficially impact the development of formula-fed infants.

Previous studies have demonstrated that infant formulas supplemented with MOS (8–10 g/L) and with or without probiotics support age-appropriate growth, are safe and well tolerated (13, 14). Previous studies also showed a bifidogenic effect of formula with MOS (6–10 g/L) and probiotics (13–17), and 1 study reported increased secretory IgA (sIgA) with a formula containing MOS (8 g/L) and *Bifidobacterium* (*B*.) *lactis* (14). The only trial that has studied MOS (10 g/L) without a probiotic found bifidobacteria counts in the MOS only group that were between (but not significantly different from) the control group and another experimental group receiving formula with MOS and 2 probiotics (13). The current trial studied the addition of MOS alone at a lower dose (7.2 g/L) and the herein reported findings are the secondary endpoints of a randomized controlled trial, whose coprimary endpoints (weight gain and stool consistency) have been published separately (18). For the present report also including a companion study of human milk-fed infants (HFI), we hypothesized that infants receiving a formula with MOS at 7.2 g/L would have an overall gut microbiota composition closer to that observed in HFI and an improved intestinal immune response compared with their control peers.

## Methods

### Study design and population

A randomized, double-blind, controlled trial of 2 formula-fed groups and a prospective, observational companion study of HFI were conducted between January 2016 and December 2018 in the Asian Hospital and Medical Center, Muntinlupa City, Philippines, in compliance with the Declaration of Helsinki and the International Conference on Harmonization Guidelines for good clinical practice. Prior to enrollment, written informed consent was obtained from the parent(s)/legally accepted representative (hereafter called parents) of the infants. The studies were approved by the Asian Hospital Institutional Review Board, Muntinlupa City, Philippines, and are registered at clinicaltrials.gov as NCT02670863 and NCT03387124.

Major inclusion criteria were: *1*) healthy term, singleton birth (37–42 weeks of gestation), *2*) postnatal age of 21–26 d at enrollment, *3*) weight-for-length and head circumference-for-age *z*-scores between –3 and +3 according to the WHO Child Growth Standards at enrollment, and *4*) exclusive consumption and tolerance of intact protein cow milk formula for ≥3 d prior to enrollment (for formula-fed infants) or exclusive human milk feeding and infant's parents decided to continue exclusive human milk feeding up to the age of 6 mo (for HFI). Major exclusion criteria included: *1*) evidence of major congenital malformation, *2*) significant prenatal and/or serious postnatal disease before enrollment (by medical decision), *3*) admission to a neonatal intensive care unit except for admission for jaundice phototherapy, *4*) parents aged <18 y, or *5*) prior participation in another clinical trial since birth.

Eligible formula-fed infants were randomly assigned to either the control group (CG) or experimental group (EG) stratified by delivery mode and sex and were fed with the formulas from enrollment/baseline (aged 0.75 mo) until 6 mo of life. Randomization was carried out using a dynamic allocation algorithm with Medidata Balance. Parents, investigators, and study support staff were blinded to the identity of the study formulas using a total of 6 codes (3 per formula group) that were provided by the study sponsor. For all feeding groups, fecal samples were collected at the age of 0.75 (baseline), 2.5, and 4 mo. At the age of 2.5 mo, fecal samples were collected only in a subset of infants (∼75/feeding group).

### Interventions

The control formula was composed of 65% intact whey protein (enriched in α-lactalbumin) and 35% casein protein ratio, carbohydrates consisting of 100% lactose, and a vegetable oil blend high in *sn-2* palmitate (66.2 kcal/100 mL reconstituted formula). The experimental formula was identical to the control formula except for the addition of the MOS ingredient providing a total of 7.2 g oligosaccharides per liter of reconstituted formula. MOS were enzymatically derived from lactose-rich whey permeate. The oligosaccharide profile consisted of mainly galacto-oligosaccharides with an average degree of polymerization of 2.88, in addition to small amounts of sialylated-oligosaccharides. Approximately 60 mg/L of the total oligosaccharides in the experimental formula were sialylated. Parent(s) were advised to feed the study formulas to their infants as they deemed appropriate, based on the infant's appetite, age, and weight. Both formulas were light colored powders with a slightly granular appearance and the same odor and taste characteristics. They were produced at the Wyeth Nutrition factory in Askeaton, Co. Limerick, Ireland.

### Fecal DNA extraction and 16S ribosomal RNA gene sequencing

DNA isolation, including vigorous bead-beating steps, was performed as described previously (19). Barcoded amplicons from the V3–V4 region of 16S ribosomal RNA (rRNA) genes were generated using a 2-step PCR and according to previously described methods (19). Library preparation was performed at BaseClear BV (Leiden, The Netherlands). PCR products were checked on a Bioanalyzer (Agilent) and quantified. This was followed by multiplexing, clustering, and sequencing on an Illumina MiSeq with the paired-end (2×) 300 bp protocol and indexing. The sequencing run was analyzed with the Illumina CASAVA pipeline (v1.8.3) with demultiplexing based on sample-specific barcodes. Sequence reads of too low quality (only “passing filter” reads were selected) and reads containing adaptor sequences or PhiX control were discarded from the raw sequencing data. On the remaining reads, a quality assessment was performed using FastQC version 0.10.0 (Babraham Bioinformatics).

### 16S rRNA gene sequence analysis and statistics

16S rRNA gene sequences were analyzed using a workflow based on Qiime 1.8 (20). On average, 29,570 (range 3308–148,882) sequences were obtained per sample to define their taxonomic profiles. We performed operational taxonomic unit (OTU) clustering (open reference), taxonomic assignment, and reference alignment with the pick_open_reference_otus.py workflow script of Qiime, using default parameters, *uclust* as the clustering method (97% identity), and GreenGenes v13.8 as the reference database for taxonomic assignment. Reference-based chimera removal was done with Uchime (21). The RDP classifier version 2.2 was performed for taxonomic classification (22). All sequences annotated as *Bifidobacterium* were individually assigned to species and subspecies based on signature sequences, as previously described (4). Statistical tests were performed as implemented in SciPy (https://www.scipy.org/), downstream of the Qiime-based workflow. At each time point, we tested for between-group differences in α-diversity [Faith's phylogenetic diversity (PD_whole tree), observed species index, Shannon diversity index; all based on 10 rarefactions] and β-diversity (weighted UniFrac based on 10 rarefactions; for each infant in a group we calculated the average distance to all infants in another group) using the nonparametric Kruskal–Wallis test with Dunn's post hoc test, as implemented in Graphpad Prism 5.01. Between-group differences of single taxa at each time point were assessed using nonparametric tests. For comparisons of >2 groups, the nonparametric Kruskal–Wallis test with false discovery rate correction (*P* < 0.05) was applied. These bivariate analyses were performed on a selection of taxa important in the studied age range. For the taxa, significantly different between the 3 feeding groups at *P* < 0.05, pairwise comparisons were made with Dunn's post hoc test.

To assess the differences in microbiota compositions between feeding groups, we performed multivariate redundancy analyses (RDAs) as assessed by 16S rRNA gene sequencing in Canoco version 5.12 using default settings of the analysis type “Constrained” (23). Relative abundance values of OTUs or species were used as response data, and metadata as the explanatory variable. For visualization purposes, families or genera, rather than OTUs, were plotted as supplementary variables. Variation explained by the explanatory variables corresponds to the classical coefficient of determination (R2) and was adjusted for degrees of freedom (for explanatory variables) and the number of cases. Canoco determines RDA significance by permutating (Monte Carlo) the sample status. Per time point and sample set, confounding factors were first identified by RDA. Statistically significant confounders were included as covariates in subsequent analyses. Hence, partial RDA was employed to correct for covariance where relevant, covariates were first fitted by regression and then partialled out (removed) from the ordination.

### Pathogenic bacteria species by qPCR

Detection and quantification of selected genes of pathogenic bacteria species including *Clostridioides difficile* (*C. difficile*) 16S and toxB, *Clostridium perfringens*(*C. perfringens*), *Klebsiella* (*K*.) *pneumonia*, enteropathogenic *Escherichia coli* (EPEC), enterotoxigenic *Escherichia coli* (ETEC) heat-labile toxin (LT) and ETEC heat-stable toxin (ST),*Salmonella* species, *Campylobacter jejuni* (*C. jejuni*), and *Campylobacter coli*(*C. coli*) were done at baseline and at the age of 2.5 and 4 mo with isolated DNA from fecal samples according to validated qPCR assays described in the **Supplemental Methods**.

### Fecal pH, calcium, and organic acid analysis

Fecal pH was assessed using an electrode-fitted pH meter after suspending 0.5 g (fresh weight) of fecal sample in 2 mL milliQ water. For organic acid analysis (lactate, acetate, propionate, butyrate, isobutyrate, valerate, isovalerate), fecal samples were prepared according to a modified and previously described method (24). Fecal calcium excretion was quantified using inductively coupled plasma atomic emission spectroscopy as previously described (25) (see also Supplemental Methods). All parameters were analyzed at baseline and at the age of 4 mo.

### Fecal markers of intestinal immune and barrier function

Fecal sIgA, lipocalin, calprotectin, α-1 antitrypsin, pancreatic elastase, and myeloperoxidase were analyzed at baseline and age 4 mo using commercially available ELISA kits according to the manufacturer's instructions and as indicated in the Supplemental Methods. All the formula-fed infants providing a stool sample at the age of 2.5 mo (*n* = 75/group) received an oral poliovirus vaccine (OPV) at the age of 2 mo and fecal OPV-specific IgA and IgG were determined using ELISA as detailed in the Supplemental Methods. Only 15 HFI received OPV at 2 mo of life; therefore, HFI were not included in the analysis on vaccine response.

### Additional statistical analysis

Continuous variables (qPCR counts, fecal pH, organic acids, calcium excretion, and fecal biomarkers) were analyzed among the groups using linear models including corresponding baseline value, infant sex, and mode of delivery as covariates. For the qPCR counts analysis, antibiotic treatment (yes/no) was included as an additional covariate. For organic acids, if 50% of the infants in any feeding group had values below the detection limit, models were done with the detection limit divided by 2 for the values below the detection limit. Data were expressed as grams of fecal dry weight. Categorical variables (presence of qPCR targets) were analyzed using log-binomial regression model. Skewed data were analyzed on log-scale. Statistical model assumptions (normality, homogeneity of variances) were met for all applied statistical methods. Baseline education characteristics were compared using Fisher's exact test. Associations between bifidobacteria and sIgA or calcium excretion were assessed by Spearman correlation tests. All analyses were done using SAS version 9.3 and *P* values < 0.05 were considered significant. This manuscript reports the secondary endpoints of a study for which the sample size calculation was based on the 2 coprimary endpoints, growth and stool consistency (18); therefore, no sample size calculation is available for the herein reported endpoints.

## Results

### Participants

**FIGURE 1:**
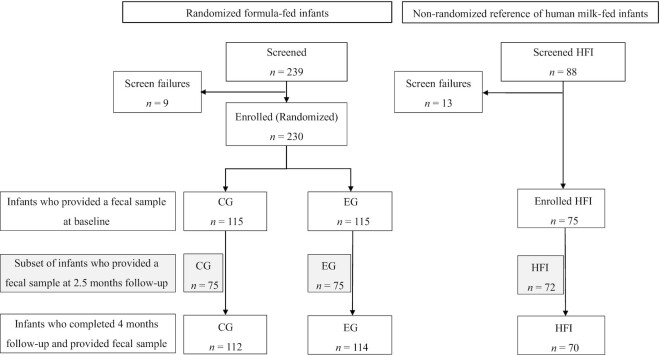
Subject disposition for the randomized controlled trial with formula-fed infants and the companion study with human milk-fed infants serving as the reference group. CG, control group; EG, experimental group; HFI, human milk-fed infants.

Out of 239 formula-fed infants assessed for eligibility, 230 infants were enrolled and randomly assigned to either CG or EG (*n* = 115 in both groups; **Figure 1**). In CG and EG, 112 and 114 infants, respectively, completed the 4 mo follow-up and provided fecal samples. In HFI, 88 infants were screened. Seventy-five infants were enrolled (13 screening failures) and 70 infants completed the 4 mo follow-up providing fecal samples (Figure 1). The baseline characteristics of the enrolled infants were comparable between the 3 groups except for a significantly higher percentage of mothers who had completed college in EG and CG compared with HFI (**Table 1**).

**TABLE 1:** Baseline characteristics of participating infants and parental education level

Characteristics^1^	CG (*n* = 112)	EG (*n* = 114)	HFI (*n* = 70)
Age at enrollment, d	23.3 ± 1.7	23.1 ± 1.7	23.7 ± 1.8
Infant sex, % male	51.8	51.8	53.3
Gestational age, wk	38.7 ± 0.9	38.7 ± 1.1	38.8 ± 1.3
Type of delivery, % vaginal	82.1	83.3	81.3
Weight, kg	3.8 ± 0.4	3.8 ± 0.4	3.9 ± 0.4
Length, cm	52.1 ± 1.4	52.0 ± 1.5	51.1 ± 1.6
Head circumference, cm	35.7 ± 1.0	35.8 ± 1.0	36.0 ± 0.9
Mother education,^2^ % completed college	33.0	25.4	10.7
Father education, % completed college	22.3	17.5	13.5

1 Values are means ± SD unless otherwise specified.

2 Significantly higher in CG and EG than in HFI (*P* < 0.05) based on Fishers exact test.

CG, control group; EG, experimental group; HFI, human milk-fed infants.

### Supplementation with MOS shifts the gut microbiota composition towards that of HFI

**FIGURE 2:**
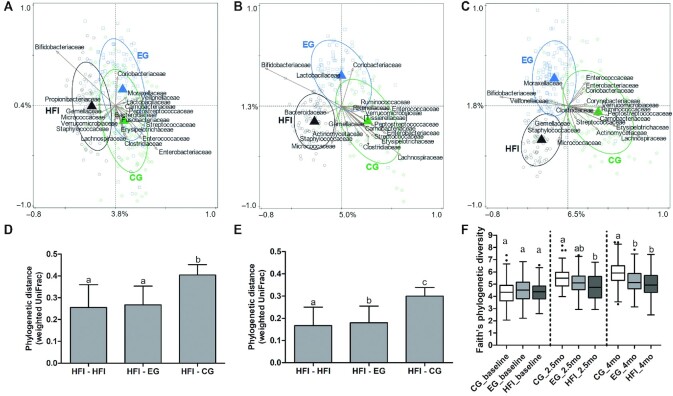
Comparison of the gut microbiota composition for the 3 feeding groups at baseline (age 0.75 mo), and aged 2.5 and 4 mo. CG, *n* = 112; EG, *n* = 114; HFI, *n* = 70 except at the age of 2.5 mo where CG and EG, *n* = 75; HFI, *n* = 72. **Panels A–C:** Redundancy analysis on the operational taxonomic unit (OTU) level at baseline (age 0.75 mo), and aged 2.5 and 4 mo. OTUs were used as response data and feeding was explanatory data, the bacterial families that contributed most were plotted as labelled arrows. The covariance attributable to confounders was first fitted by regression and then partialled out (removed) from the ordination. Ellipses represent 66% CIs from centroids. The (unadjusted) variation explained is indicated on the axes. A: baseline; variation explained by feeding was 3.5%, *P* = 0.002 (covariates: delivery mode and gender). B: 2.5 mo; variation explained by feeding was 5.5%, *P* = 0.002 (covariates: delivery mode, antibiotic treatment (yes/no), and gender). C: 4 mo; variation explained by feeding was 7.7%, *P* = 0.002 [covariates: delivery mode, antibiotic treatment (yes/no), and gender]. **Panels D–E:** Phylogenetic distances (weighted UniFrac; mean with SD) within HFI, and between HFI and the formula-fed groups at the age of 2.5 (D) and 4 mo (E). Feeding groups were compared by the Kruskal–Wallis test followed by pairwise comparisons with Dunn's posthoc test. Bars without a common superscript letter are significantly different from each other (*P* < 0.05 based on Dunn's post hoc test). **Panel F:** Faith's phylogenetic diversity index at baseline (age 0.75 mo), and aged 2.5 and 4 mo. Box plots show the median and 25th and 75th percentiles with Tukey whiskers and outliers as individual data points. Feeding groups were compared by the Kruskal–Wallis test followed by pairwise comparisons with Dunn's post hoc test. Box plots of the same time point without a common superscript letter are significantly different from each other (*P* < 0.001 based on Dunn's post hoc test). CG, control group; EG, experimental group; HFI, human milk-fed infants.

At baseline, no difference in microbiota composition between CG and EG was observed (*P* = 0.27). Including HFI, RDA analysis showed significance (variation explained 3.5%, *P* = 0.002; **Figure 2**A) indicating that CG and EG were similar, and distant from HFI. HFI were associated with higher relative abundances of *Bifidobacteriaceae*, whereas the formula-fed groups were associated with higher relative abundances of e.g. *Enterobacteriaceae* and *Streptococcaceae*. However, baseline Faith's phylogenetic diversity was similar for the 3 feeding groups (Figure 2F). Additional α-diversity indexes showed similar results, except a lower Shannon diversity in HFI (**Supplemental Figure 1**A and 1B).

Aged 2.5 and 4 mo, a clear difference was found between EG and CG (RDA, variation explained 2.4% and 5.5%, respectively, *P* = 0.002 for both). Analyzing the 3 feeding groups together, EG was positioned between CG and HFI (Figure 2B and C) and, taking into account the difference of variation explained between the 2 RDA axes, EG was closer to HFI than CG. Only EG and HFI were associated with higher proportions of *Bifidobacteriaceae*. CG was for example associated with higher proportions of *Lachnospiraceae*. Interestingly, *Staphylococcaceae* and *Micrococcaceae*, 2 typical taxa of the skin microbiota, were the main contributors to the separation between HFI and formula-fed infants (Figure 2C), possibly explained by the close contact with the maternal skin during suckling. Average phylogenetic distances between the samples (weighted UniFrac) from the different feeding groups at 2.5 and 4 mo showed that CG and HFI were more distant than EG and HFI (Figure 2D and E); as expected, this difference was not observed at baseline (data not shown). At the age of 4 mo, the microbial diversity within samples as assessed by Faith's phylogenetic diversity index was higher in CG than in EG and HFI (*P* < 0.001), with no difference between EG and HFI. A similar pattern was observed at 2.5 mo (Figure 2F). Additional α-diversity indexes showed significant differences between EG and HFI, yet indexes were significantly lower in EG compared with CG with the difference of the means between EG and HFI numerically smaller than that between CG and HFI, confirming the robustness of the observation (Supplemental Figure 1A and 1B).

**FIGURE 3:**
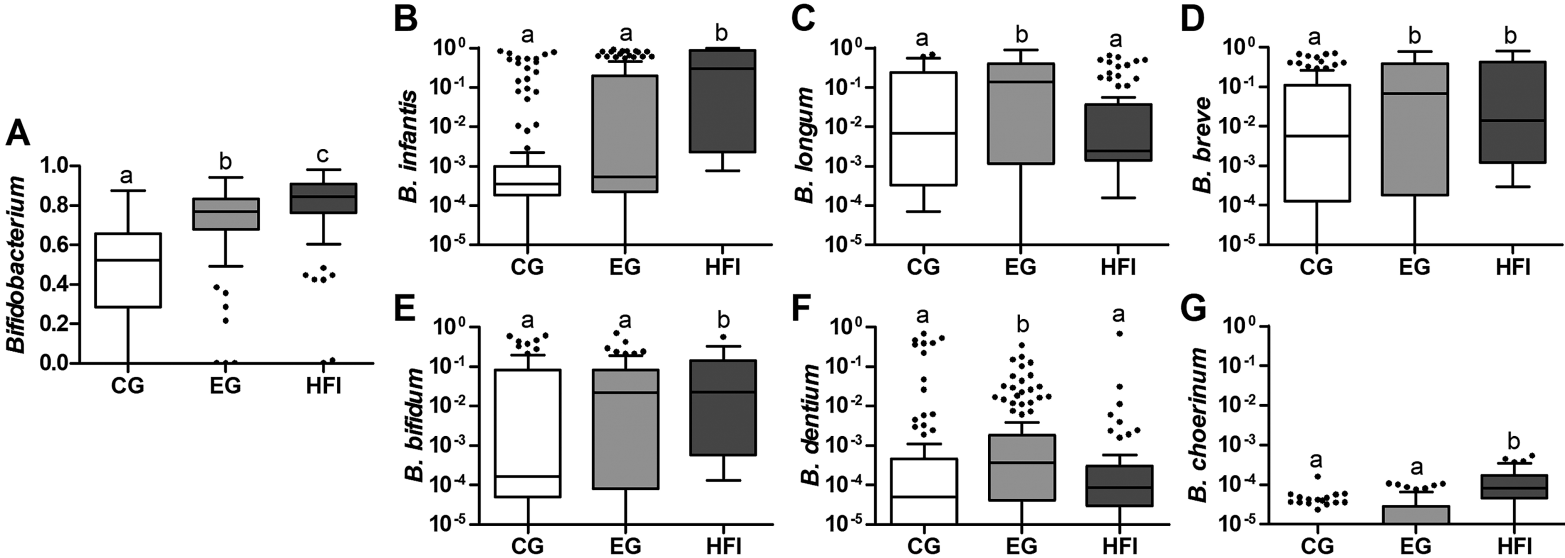
Relative abundance of genus *Bifidobacterium* (panel A) and different *Bifidobacterium* (sub-)species (panels B–G) at the age of 4 mo in the 3 feeding groups. Box plots show the median and 25th and 75th percentiles with Tukey whiskers and outliers as individual data points. Feeding groups were compared by the Kruskal–Wallis test and false discovery rate correction for multiple testing, followed by pairwise comparisons with Dunn's post hoc test. Box plots without a common superscript letter are significantly different from each other (*P* < 0.05 based on Dunn's post hoc test). For detailed mean and median values of the relative abundance of different *Bifidobacterium* species, see Supplemental Table 1. CG, *n* = 112; EG, *n* = 114; HFI, *n* = 70. CG, control group; EG, experimental group; HFI, human milk-fed infants.

Aged 2.5 and 4 mo, the relative abundance of *Bifidobacterium* was higher in EG than in CG (*P* < 0.001) and numerically more similar to HFI (although still significantly lower; *P* < 0.001; **Figure 3**A; **Supplemental Figure 2**). *Bifidobacterium* species analysis at 4 mo showed that *B. longum* subsp. *infantis,B. bifidum*, and *B. choerinum* were significantly higher in HFI compared with both formula groups (hereafter collectively named FF). Compared with CG, *B. longum* subsp. *longum,B. breve*, and *B. dentium* were stimulated in EF (*P* < 0.05; Figure 3B–G; **Supplemental Table 1**). The relative abundance of *Lactobacillus* was higher in EG compared with CG (*P* < 0.01), whereas HFI were between EG and CG, but statistically not different from them. In EG, the abundance of unclassified *Peptostreptococcaceae*, a family to which *C. difficile* belongs, was lower compared with CG (*P* < 0.001) and similar to HFI (**Supplemental Table 2**).

### Supplementation with MOS shifts the gut microbiota composition of caesarean-born infants towards that of vaginally born HFI

**FIGURE 4:**
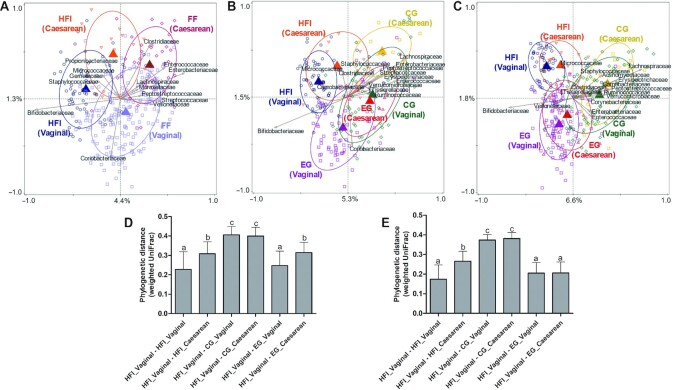
Comparison of the gut microbiota composition for the 3 feeding groups at baseline (age 0.75 mo), and the age of 2.5 and 4 mo stratified by delivery mode. CG, *n* = 112; EG, *n* = 114; HFI, *n* = 70 except aged 2.5 mo where CG and EG, *n* = 75; HFI, *n* = 72. **Panels A–C:** redundancy analysis on the operational taxonomic unit (OTU) level at baseline (age 0.75 mo), and the age of 2.5 and 4 mo. OTUs were used as response data and feeding*delivery mode was explanatory data, the bacterial families that contributed most were plotted as labelled arrows. The covariance attributable to confounders was first fitted by regression and then partialled out (removed) from the ordination. Ellipses represent 66% CIs from centroids. The (unadjusted) variation explained is indicated on the axes. A: baseline; variation explained by feeding*delivery mode was 5.1%, *P* = 0.002 (covariate: gender). B: 2.5 mo; variation explained by feeding*delivery mode was 6.5%, *P* = 0.002 [covariates: antibiotic treatment (yes/no)]. C: 4 mo; variation explained by feeding*delivery mode was 7.9%, *P* = 0.002 [covariates: gender and antibiotic treatment (yes/no)]. **Panels D–E:** phylogenetic distances (weighted UniFrac) between HFI-vaginal and the other feeding*delivery mode groups at the age of 2.5 (D) and 4 mo (E). Feeding groups were compared by the Kruskal–Wallis test followed by pairwise comparisons with Dunn's post hoc test. Bars without a common superscript letter are significantly different from each other (*P* < 0.05 based Dunn's post hoc test). CG, control group; EG, experimental group; FF, formula-fed; HFI, human milk-fed infants.

Mode of delivery influenced infant gut microbiota composition at all time points, with a decreasing effect size over time [RDA, variation explained = 1.7% (*P* = 0.002) at baseline, 0.9% (*P* = 0.002) at 2.5 mo, and 0.5% (*P* = 0.004) at 4 mo]. Hence, the interaction of delivery mode with feeding was investigated. At baseline, the 4 relevant groups (HFI*vaginal, HFI*caesarean, FF*vaginal, and FF*caesarean) showed significant gut microbiota composition differences (variation explained = 5.1%, *P* = 0.002; **Figure 4**A). Vaginal birth was associated with higher relative abundances of e.g. *Bifidobacteriaceae* and *Coriobacteriaceae*, whereas caesarean birth was associated with higher relative abundances of e.g. *Clostridiaceae, Enterobacteriaceae*, and *Propionibacteriaceae*. At 2.5 and 4 mo, RDA ordinations with 6 groups (HFI*vaginal, HFI*caesarean, CG*vaginal, CG*caesarean, EG*vaginal, and EG*caesarean) tended to separate the groups (shown as centroids) mainly on feeding along the horizontal axis (with more variation explained), and on delivery mode along the vertical axis (Figure 4B and C). Notably, at 2.5 mo, the EG*vaginal centroid was already the closest to the HFI*vaginal centroid as indicated by the overlapping ellipses, whereas the EG*caesarean centroid was nearly superimposed with the CG*vaginal centroid and far from the CG*caesarean centroid (Figure 4B). At 4 mo, the gut microbiota composition of both the EG vaginally and caesarean-born infants shifted towards that of HFI, with *Bifidobacteriaceae* as the main driver (Figure 4C). The average weighted UniFrac distances between groups confirmed that at 2.5 mo EG*vaginal was the only group close to HFI*vaginal, even closer than the HFI*caesarean was to HFI*vaginal. The CG groups were clearly the most distant from HFI*vaginal, and EG*caesarean and HFI*caesarean were in intermediate positions. (Figure 4D). At 4 mo, the same situation was observed except that EG*caesarean moved close to HFI*vaginal, similarly to EG*vaginal (Figure 4E). *Bifidobacterium* abundance in EG*caesarean was comparable with that in EG*vaginal at the age of 2.5 and 4 mo (**Supplemental Figure 3;Supplemental Table 3**).

### Pathogenic bacteria species

**FIGURE 5:**
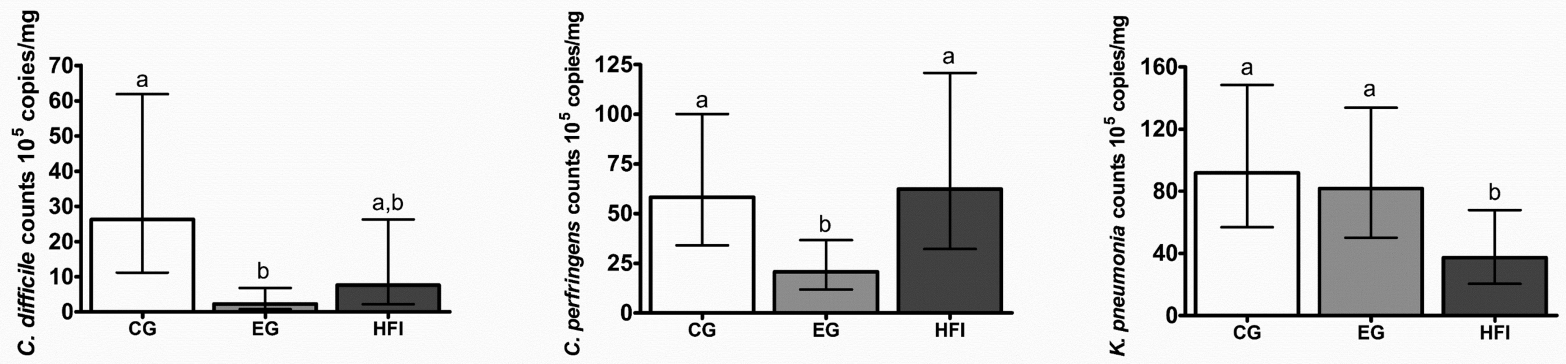
Counts of selected opportunistic bacterial pathogens in the 3 feeding groups at the age of 4 mo analyzed by qPCR. *Clostridioides difficile* (*C. difficile*) counts are based on 16S target. Data is presented as geometric LS means with the 95% CI as whiskers. Bars without a common superscript letter are significantly different from each other (*P* < 0.05) based on a linear model adjusted for baseline counts, gender, mode of delivery, antibiotic treatment (yes/no), and visit. CG, *n* = 60; EG, *n* = 48; HFI, *n* = 32 for *C. difficile*; CG, *n* = 107; EG, *n* = 102; HFI, *n* = 55 for *Clostridium perfringens*(*C. perfringens*); CG, *n* = 110; EG, *n* = 114; HFI, *n* = 65 for *Klebsiella* (*K*.) *pneumonia*. CG, control group; EG, experimental group; HFI, human milk-fed infants.

Compared with CG, counts of *C. difficile* (based on 16S target) and *C. perfringens* in EG were ∼90% (*P <*0.001) and ∼65% (*P <*0.01) lower at the age of 4 mo, respectively (**Figure 5**). HFI had *C. difficile* counts not significantly different from the formula groups with a smaller numerical difference between the means of HFI and EG than between HFI and CG. *C. perfringens* counts in HFI were not different from CG and higher than in EG (*P =* 0.01). *K. pneumonia* counts were not different between the formula groups, but lower in HFI (*P <*0.05 compared with both formula groups). The low prevalence of EPEC, ETEC LT or ST,*C. difficile* toxB,*Salmonella* species, *C. jejuni*, and *C. coli*, did not allow for any statistical analysis on counts. However, pooling the prevalence of some of these targets at the age of 2.5 and 4 mo allowed calculation of ORs (**Supplemental Table 4**). The odds of having *C. difficile* 16S or toxB (both *P <* 0.001),*C. perfringens*, or *K. pneumonia*(both *P <*0.05) were lower in EG compared with CG. HFI had reduced odds for EPEC (*P <* 0.001) and *K. pneumonia*(*P <* 0.05) compared with formula groups.

### Fecal pH, organic acids, and calcium excretion

At the age of 4 mo, fecal pH in EG was ∼1 pH unit lower than in CG [Geometric (geom.) LS mean (95% CI): 5.6 (5.5, 5.8) compared with 6.6 (6.4, 6.8), respectively; *P* < 0.001] and only about 0.2 units higher than in HFI [5.4 (5.2, 5.6); *P* < 0.05; data not illustrated]. In line with this, concentrations of total fecal organic acids (*P* < 0.01), lactate (*P* < 0.001), and acetate (*P <* 0.05) in EG were higher than in CG and comparable to HFI (**Table 2**). HFI had higher concentrations of total fecal organic acids (*P* < 0.05) and lactate (*P* < 0.001) than CG whereas acetate was not different from the formula-fed groups. Concentrations of butyrate, propionate, isovalerate, isobutyrate (all *P* < 0.001), and valerate (*P* < 0.01) were lower in EG than in CG. In HFI, the concentrations of these organic acids were significantly lower than in the formula-fed groups (except for valerate which was not different between HFI and EG). Calcium excretion in EG [geom. LS mean (95% CI): 447.3 (395.2, 506.3) μmol/g] was similar to HFI [450.5 (379.4, 535.0) μmol/g]; both were lower than in CG [623.2 (550.3, 705.8) μmol/g*; P* < 0.005; data not illustrated]. Interestingly, lower calcium excretion was correlated with higher *Bifidobacterium* relative abundance (*r* = –0.4272, *P* < 0.001) at the age of 4 mo considering the whole study population (data not illustrated).

**TABLE 2:** Total concentration of fecal organic acids and individual organic acids at the age of 4 mo

Bacteria species	CG (*n* = 112)	EG (*n* = 114)	HFI (*n* = 70)
Total organic acids,^1^ mg/kg	342.3 (300.0, 390.6)^a^	437.0 (383.0, 498.7)^b^	417.3 (352.1, 494.5)^b^
Acetate, mg/kg	173.8 (145.0, 208.4)^a^	231.9 (193.4, 277.9)^b^	206.0 (164.5, 258.0)^a,b^
Lactate, mg/kg	15.4 (11.0, 21.8)^a^	109.3 (78.4, 152.4)^b^	128.0 (84.2, 194.3)^b^
Propionate, mg/kg	36.3 (23.2, 56.9)^a^	14.9 (9.5, 23.5)^b^	1.1 (0.5, 2.5)^c^
Butyrate, mg/kg	30.3 (19.6, 46.8)^a^	5.4 (3.5, 8.3)^b^	1.9 (1.1, 3.1)^c^
Valerate, mg/kg	1.1 (0.8, 1.6)^a^	0.6 (0.4, 0.9)^b^	0.4 (0.3, 0.7)^b^
Isobutyrate, mg/kg	5.2 (3.7, 7.2)^a^	1.0 (0.7, 1.4)^b^	0.6 (0.4, 0.9)^c^
Isovalerate, mg/kg	6.9 (5.0, 9.6)^a^	1.2 (0.9, 1.7)^b^	0.6 (0.4, 0.9)^c^

1 Values are geometric LS means with 95% CI in parentheses. Linear models controlling for baseline value, infant sex, and mode of delivery were used for pairwise comparisons. Values without a common superscript letter are significantly different from each other (*P* < 0.05). Data is expressed per fecal dry weight. CG, control group; EG, experimental group; HFI, human milk-fed infants.

### Fecal markers of intestinal immune and barrier function

**FIGURE 6:**
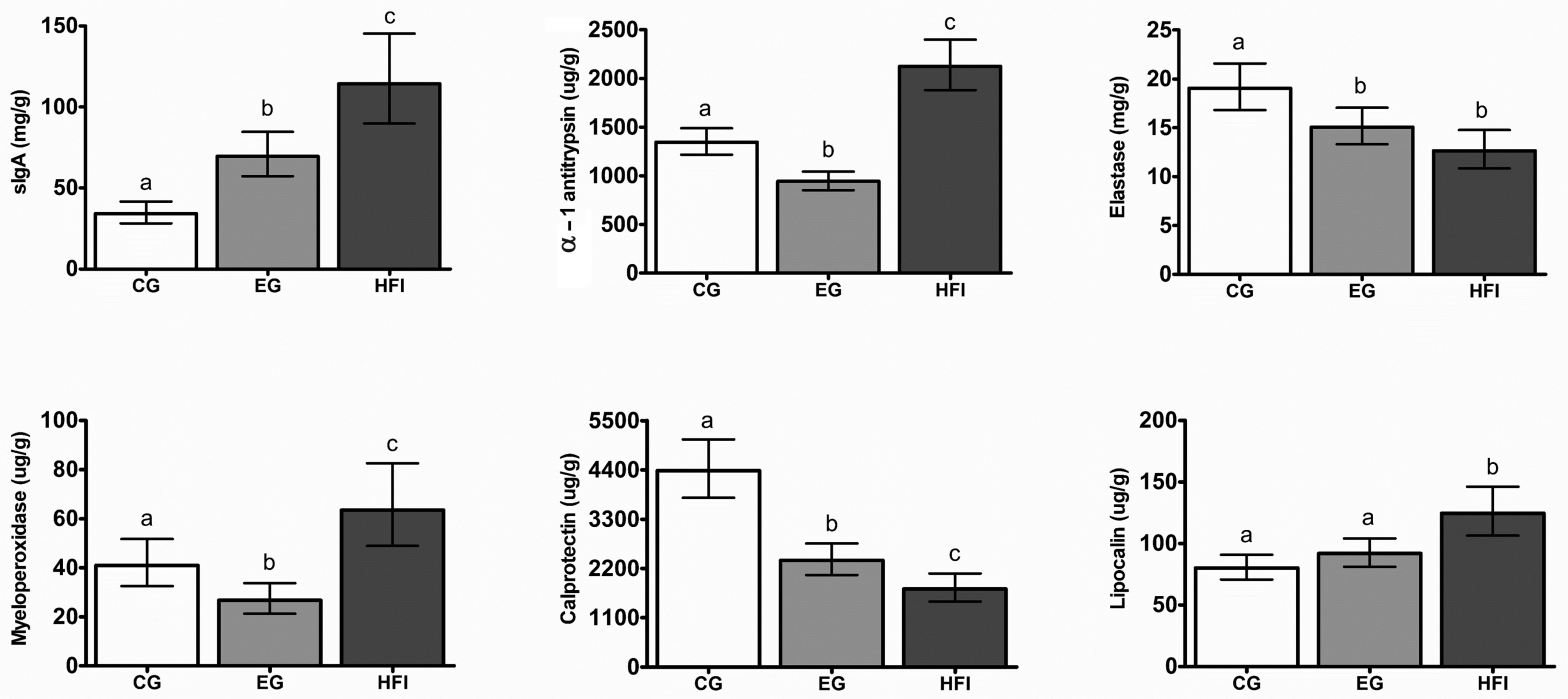
Concentration of fecal markers of intestinal immunity, permeability, and inflammation at the age of 4 mo. Data is presented as geometric LS-means with the 95% CI as whiskers and expressed per fecal dry weight. Bars without a common superscript letter are significantly different from each other (*P* < 0.05) based on a linear model adjusted for baseline value, infant sex, and mode of delivery. CG, *n* = 112; EG, *n* = 114; HFI, *n* = 70; except for α-1 antitrypsin where CG, *n* = 111; EG, *n* = 112 and myeloperoxidase where CG, *n* = 106; EG, *n* = 110. CG, control group; EG, experimental group; HFI, human milk-fed infants; sIgA, secretory IgA.

Concentrations of fecal markers of intestinal immunity, permeability, and inflammation at the age of 4 mo are shown in **Figure 6**. Notably, the sIgA concentration in EG was twice as high compared with CG (*P* <0.001). As expected, HFI had the highest sIgA concentration (*P* < 0.001). Aged 4 mo, the sIgA concentration was positively correlated with *Bifidobacterium* abundance in the whole study population (*r* = 0.335, *P* < 0.001; data not illustrated). Concentrations of α-1 antitrypsin, calprotectin (both *P* < 0.001), and elastase and myeloperoxidase (both *P* < 0.01) were lower in EG compared with CG. Lipocalin concentration tended to be higher in EG compared with CG (*P =* 0.09). In HFI, concentrations of α-1 antitrypsin, myeloperoxidase, and lipocalin (all *P* < 0.001) were highest. The numerical difference of the means for α-1 antitrypsin and myeloperoxidase was smaller for HFI versus CG than for HFI versus EG, whereas for lipocalin it was smaller for HFI versus EG compared with HFI versus CG. Concentrations of elastase (*P* < 0.001 versus CG; *P =* 0.06 versus EG) and calprotectin (*P* < 0.001) were lowest in HFI with the difference of the means being numerically smaller for HFI compared with EG than for HFI compared with CG. Fecal OPV-specific IgA measured at the age of 2.5 mo in a subset of infants was ∼50% higher in the EG compared with CG [geom. LS mean (95% CI): 51 (27, 98) mg/g compared with 25 (12, 50) mg/g, *P* = 0.065; data not illustrated]. Measures of fecal OPV-specific IgG at the age of 2.5 mo showed no difference between EG and CG [geom. LS mean (95%): 34 (13, 89) mg/g in EG compared with 59 (20, 172) mg/g in CG, *P* = 0.31; data not illustrated].

## Discussion

Adding MOS to infant formula had a strong effect on gut microbiota and our study is the first to demonstrate a significant increase in bifidobacteria with an infant formula supplemented with MOS alone. The previously reported increases in bifidobacteria were for infant formulas with combined probiotics and MOS (8–10 g/L) (13–17). *Bifidobacterium* species in the gastrointestinal tract of infants have been shown to metabolize galacto- or sialylated oligosaccharides. However, their ability to utilize these oligosaccharides is species or even strain specific (26, 27). We found noteworthy differences in *Bifidobacterium* (sub)-species abundances. Higher abundances of *B. dentium,B. longum* subspecies *longum*, and *B. breve* in EG compared with CG suggest that strains of these (sub)-species can metabolize oligosaccharides present in MOS; thus, gaining a competitive advantage over other (sub)-species. A previous study also found increased *B. breve* colonization in infants receiving galacto-oligosaccharide supplemented formula (5 g/L) compared with a control group (28), and *B. longum* subspecies *longum* has been shown to consume sialylated oligosaccharides in in vitro studies (29). The higher abundance of *B. choerinum, B. longum* subspecies *infantis*, and *B. bifidium* observed in HFI suggests that they gain a competitive advantage especially with fucosylated and nonfucosylated oligosaccharides found in human milk, but absent in MOS. Both *B. longum* subspecies *longum* and subspecies *infantis* are, however, expected to have the genetic makeup to use the dominant oligosaccharides (30). Interestingly, the effects of the MOS-supplemented formula on the gut microbiota in caesarean- or vaginally born infants were similar, changing the microbiota towards the composition observed in vaginally born HFI including an increase in bifidobacteria, although at a slower pace for the caesarean-born infants. This suggests that MOS helps to correct some of the well-documented dysbiosis in caesarean-born delivered infants (31), similarly to the effect reported for human milk (32, 33).

The higher concentration of lactate and acetate in EG compared with CG is likely a result of the higher bifidobacteria in EG as both are main endproducts of bifidobacteria catabolism (34). On the other hand, the higher concentrations of propionate and butyrate in CG indicate a more complex microbiota, given that they are produced by *Bacteroides* and *Firmicutes* (e.g. *Clostridium*), but not *Bifidobacterium* (35). Acetate and lactate reduce gut pH (34) which may suppress the growth of pathogenic bacteria (36) and can improve calcium availability by increasing its solubility (37). Indeed, in our study, calcium excretion was negatively correlated with bifidobacteria and lower in EG and HFI than in CG indicating improved calcium solubility and absorption in EG and HFI. Acetate might also have contributed directly to the reduced *C. perfringens* and *C. difficile* counts in EG. A recent in vivo study showed that acetate promotes the host innate responses against *C. difficile* through coordinated action on neutrophils and group 3 innate lymphoid cells (38). Additionally, sialylated oligosaccharides in MOS may have played a role by competing with the pathogens for the binding site of sialic acid on the gut epithelial cells; hence, preventing or reducing their adhesion, similarly as shown for *Salmonella* or *E. coli* strains in in vitro studies (39). The lower *C. perfringens* counts in EF compared with CG and HFI may be explained by some specific effect of galacto-oligosaccharides in MOS on *C. perfringens* growth. An enhanced epithelial barrier function against *C. difficile* toxin was found when lactobacilli were stimulated by MOS preparations (12), suggesting that the increased *Lactobacillus* abundance in EG in our study may have contributed to the reduced *C. difficile* load.

The 2-fold higher sIgA concentration in EG at the age of 4 mo is possibly linked to the increase in bifidobacteria which have been shown to interact with human immune cells and to modulate innate and adaptive immune processes (40). It has been suggested that the beneficial effects of bifidobacteria on the host's immune system are exerted via immunomodulatory functions of some of their surface-associated molecules (41, 42). Indeed, infant formula supplemented with bifidobacteria has been shown to increase fecal sIgA (43). sIgA is produced by B lymphocytes in the submucosal tissues and plays an essential role in protection from antigens, toxins, and potential pathogens (44). In addition to the sIgA produced in the infant gut, sIgA is also provided by human milk and explains why the highest sIgA concentration was found in HFI. Compared with a previous study with MOS and *B. lactis* where sIgA concentrations were ∼1.5- and 1.7-fold higher in the experimental group at the age of 3 and 6 mo, respectively (14), our study indicates that MOS alone without probiotics can also substantially increase sIgA. The extent to which MOS directly contributed to the sIgA increase is uncertain. Sialylated oligosaccharides in MOS may have contributed to the increase in sIgA through a proposed immunomodulatory action (26).

Remarkably, the concentration of OPV-specific IgA was ∼50% higher in EG suggesting an improved response to OPV in EG compared with CG. The effect is likely driven by the observed differences in the gut microbiota between the formula-fed groups. The gut microbiota may influence vaccine responses indirectly by affecting the development of T cells (45) or by its metabolic products (46), such as acetate, as indicated in mice models (47, 48). Previous studies indicate that bifidobacteria, in particular, and consequently their metabolic products could play an important role in the OPV response. Infants receiving a formula with *B. lactis* for 6 wk had increased fecal OPV-specific IgA concentrations compared with control peers (43). Furthermore, positive associations between *Bifidobacterium* abundance at the age of 4 mo and antipolio virus IgA titers (49), as well as fecal polio-specific IgA at the age of 2 y were reported (50). We did not find an effect on OPV-specific IgG, possibly because we measured the response too early at the age of 2.5 mo. IgG is only present in the gut by transduction which likely only happens after full potential production in the systemic compartment, and we did not collect any blood samples.

Fecal pancreatic elastase concentration in infants is a reliable marker of pancreatic function (51). Infants in EG had elastase concentrations comparable to those of HFI, both being lower than CG. This suggests that MOS contribute positively to metabolic homeostasis, possibly through the microbiome. In a previous study, infants fed formula supplemented with MOS, *B. lactis*, and lactoferrin also had an elastase concentration comparable with HFI and lower than the control (17). In our study, concentrations of α-1 antitrypsin and myeloperoxidase, markers of intestinal permeability or neutrophil activity in the intestinal mucosa, respectively, were higher in HFI than in the formula-fed infants as previously reported (52). Either HFI still had higher leakage from the gut mucosa or more likely, part of the measured α-1 antitrypsin and myeloperoxidase stems from human milk that passed to the infant feces as previously reported (52, 53). For both markers, it is known that they decrease during infancy (52) and our data suggests that MOS can contribute to this decline in formula-fed infants which appears to be the normal course. The results for calprotectin, a marker for gastrointestinal inflammation (54), suggest that MOS may contribute to the known downward trend of calprotectin in infants with increasing age (55, 56). Altogether, our fecal biomarker data indicates that MOS-supplemented formula may support gut maturation.

Our study has several strengths. The very low dropout rate resulted in a high number of analyzed stool samples (>100 per formula group and ∼70 in HFI). We used an approach that accurately annotated the 16S rRNA gene sequences belonging to the genus *Bifidobacterium* down to the (sub)-species level and we complemented 16S rRNA with qPCR analysis on specific pathogenic targets allowing us to analyze the overall gut microbiota, *Bifidobacterium* (sub-)species, and opportunistic bacterial pathogens. Also, selected fecal biomarkers were measured to strengthen the assessment of impact on infant gut physiology. A limitation of our study is that we did not measure the impact of the MOS intervention on systemic immunity. Considering the strong results on gut microbiota and its suggested link to immune development in infants, it would have been interesting to also analyze systemic immunity markers (e.g. plasma cytokines). However, as blood collection in infants can be challenging and has an ethical sensitivity in healthy infants, it was not considered in our study. We assessed samples up to the age of 4 mo; therefore, midlong-term effects on immune development and immune response were not captured. Maternal education was higher in the formula groups than in HFI, likely because in the Philippines, mothers with higher education are more engaged in the working environment (e.g. go back to work early); therefore, rather elect formula feeding than human milk feeding. However, we do not expect that this socioeconomic difference has affected our results.

To conclude, our study shows that the gut microbiota and intestinal immunity of formula-fed infants can be beneficially modulated by an infant formula with an oligosaccharide profile enhanced by the addition of MOS. The consumption of MOS-supplemented formula in the first 4 mo of infancy shifts the overall microbiota composition closer to that in HFI, has a strong bifidogenic effect, and reduces opportunistic pathogens which decreases a risk factor for developing diarrheal illness. This may mediate to a certain extent the observed effects on intestinal immunity, evidenced by the substantial increase in fecal sIgA and OPV-specific IgA. Supplementing infant formula with MOS is therefore a promising approach to support the development of the gut microbiota during early infancy in concert with the infant's immune development.

## Supplementary Material

nqab336_Supplemental_File

## Data Availability

Data described in the manuscript, code book, and analytic code will be made available upon request pending application and approval.

## Notes

This study was funded by Nestlé Nutrition, Société des Produits Nestlé S.A., Vevey, Switzerland.

Supplemental Methods, Supplemental Figures 1–3, and Supplemental Tables 1–4 are available from the “Supplementary data” link in the online posting of the article and from the same link in the online table of contents at https://academic.oup.com/ajcn/.

CIC and BB are joint senior authors.

Abbreviations used: *B*.,*Bifidobacterium; C. difficile,Clostridioides difficile; C. perfringens,Clostridium perfringens; C. jejuni,Campylobacter jejuni; C. coli,Campylobacter coli*; CG, control group; EG, experimental group; EPEC, enteropathogenic *Escherichia coli*; ETEC, enterotoxigenic *Escherichia coli*; FF, formula-fed; HFI, human milk-fed infants; *K*.,*Klebsiella*; LT, heat-labile toxin; MOS, bovine milk-derived oligosaccharides; OPV, oral poliovirus vaccine; OTU, operational taxonomic unit; RDA, redundancy analysis; rRNA, ribosomal RNA; sIgA, secretory IgA; ST, heat-stable toxin.
