# Supplementary material for: Term infant formula supplemented with milk-derived oligosaccharides shifts the gut microbiota closer to that of human milk-fed infants and improves intestinal immune defense: a randomized controlled trial
Source: Am J Clin Nutr. 2021 Oct 7;115(1):142–53. doi: 10.1093/ajcn/nqab336 (PMC8755036; doi:10.1093/ajcn/nqab336)
Supplement: nqab336_Supplemental_File [file nqab336_supplemental_file.zip › Suppl figures_MOS efficacy paper 21Sep2021.pptx]

## Slide 1
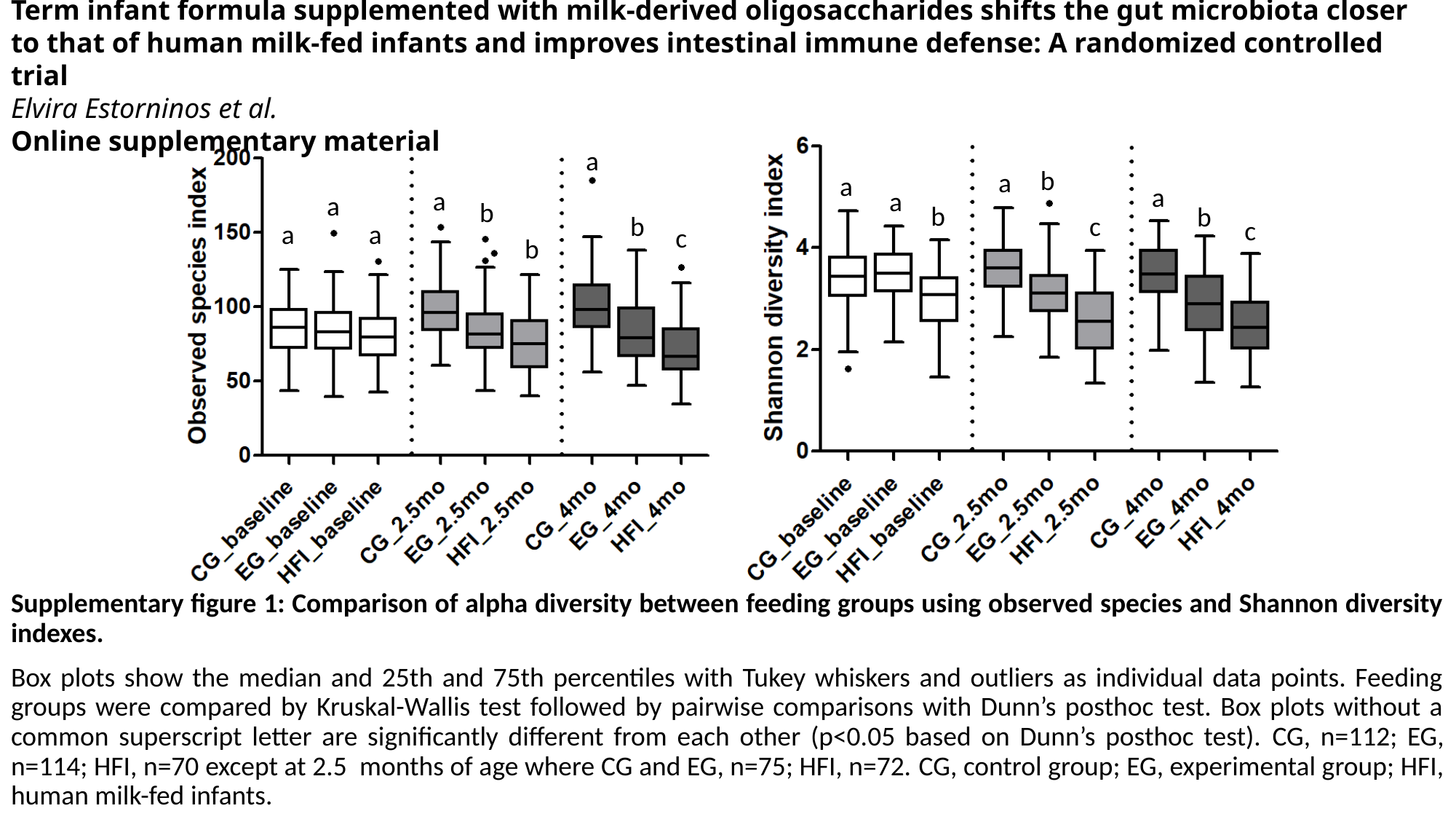

Term infant formula supplemented with milk-derived oligosaccharides shifts the gut microbiota closer to that of human milk-fed infants and improves intestinal immune defense: A randomized controlled trial
Elvira Estorninos et al.
Online supplementary material
a
b
a
a
a
a
a
a
b
b
b
c
b
c
a
a
c
b
Supplementary figure 1: Comparison of alpha diversity between feeding groups using observed species and Shannon diversity indexes.
Box plots show the median and 25th and 75th percentiles with Tukey whiskers and outliers as individual data points. Feeding groups were compared by Kruskal-Wallis test followed by pairwise comparisons with Dunn’s posthoc test. Box plots without a common superscript letter are significantly different from each other (p<0.05 based on Dunn’s posthoc test). CG, n=112; EG, n=114; HFI, n=70 except at 2.5 months of age where CG and EG, n=75; HFI, n=72. CG, control group; EG, experimental group; HFI, human milk-fed infants.

## Slide 2
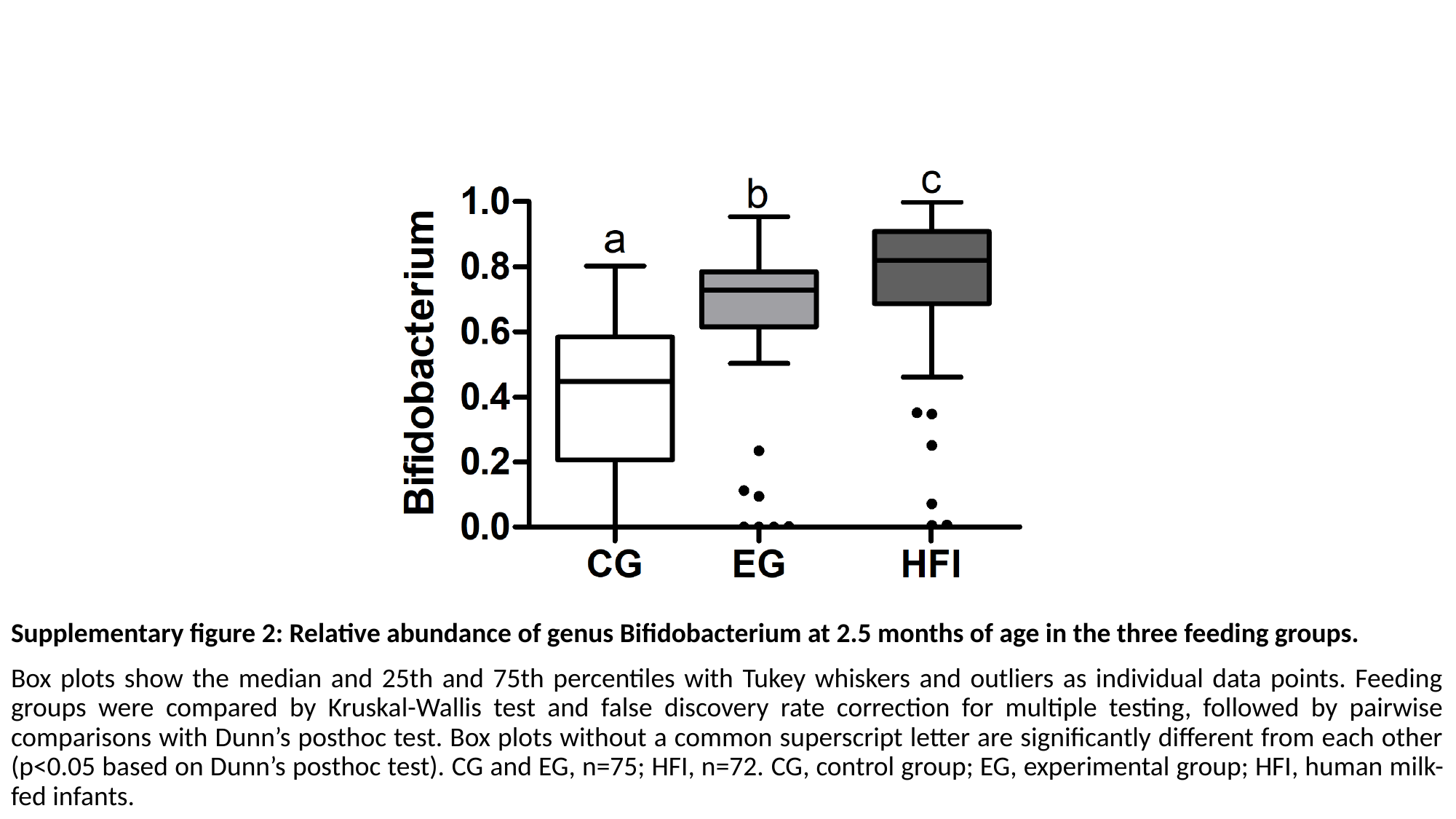

Supplementary figure 2: Relative abundance of genus Bifidobacterium at 2.5 months of age in the three feeding groups.
Box plots show the median and 25th and 75th percentiles with Tukey whiskers and outliers as individual data points. Feeding groups were compared by Kruskal-Wallis test and false discovery rate correction for multiple testing, followed by pairwise comparisons with Dunn’s posthoc test. Box plots without a common superscript letter are significantly different from each other (p<0.05 based on Dunn’s posthoc test). CG and EG, n=75; HFI, n=72. CG, control group; EG, experimental group; HFI, human milk-fed infants.

## Slide 3
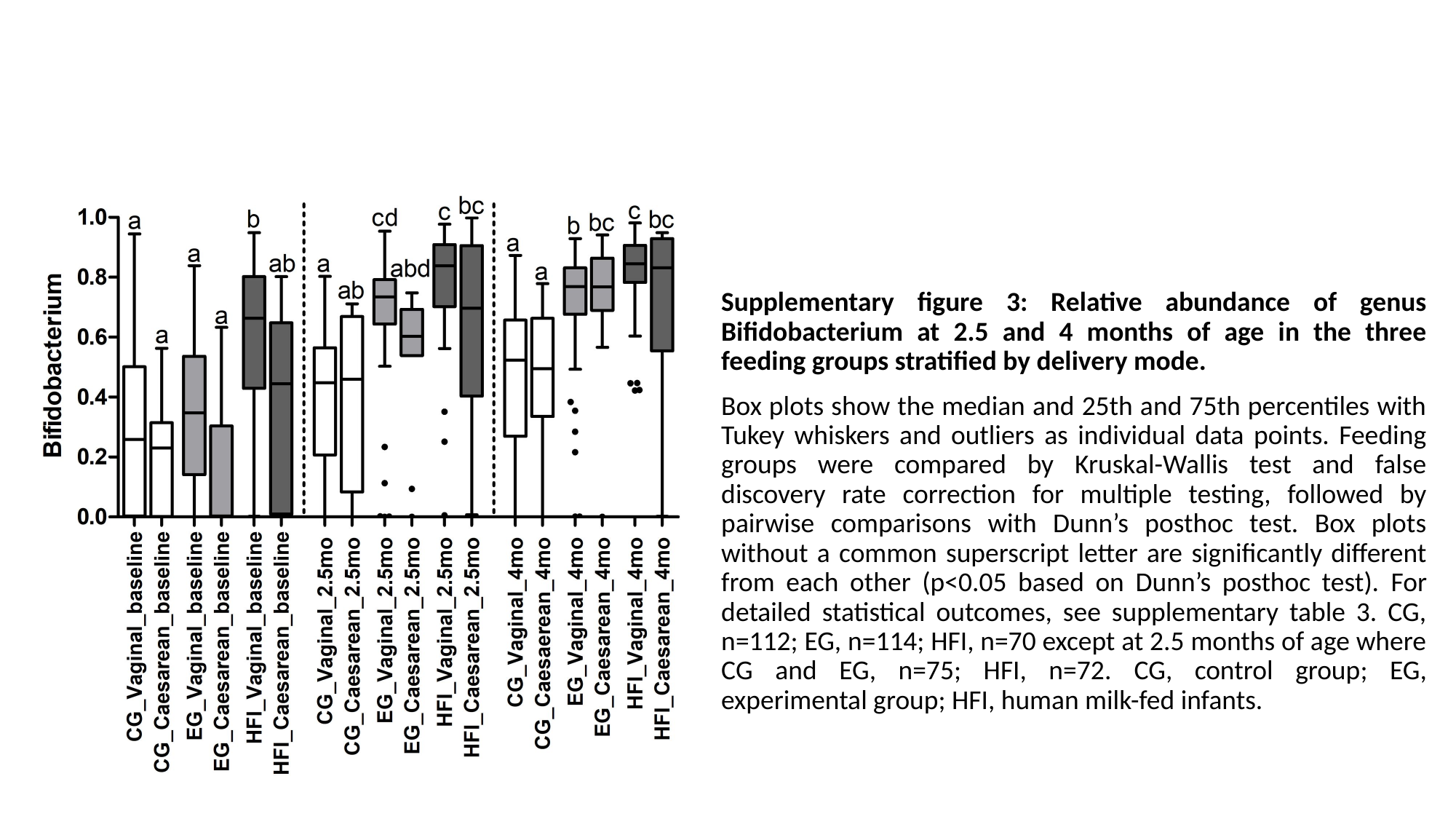

Supplementary figure 3: Relative abundance of genus Bifidobacterium at 2.5 and 4 months of age in the three feeding groups stratified by delivery mode.
Box plots show the median and 25th and 75th percentiles with Tukey whiskers and outliers as individual data points. Feeding groups were compared by Kruskal-Wallis test and false discovery rate correction for multiple testing, followed by pairwise comparisons with Dunn’s posthoc test. Box plots without a common superscript letter are significantly different from each other (p<0.05 based on Dunn’s posthoc test). For detailed statistical outcomes, see supplementary table 3. CG, n=112; EG, n=114; HFI, n=70 except at 2.5 months of age where CG and EG, n=75; HFI, n=72. CG, control group; EG, experimental group; HFI, human milk-fed infants.
