# Supplementary material for: Term infant formula supplemented with milk-derived oligosaccharides shifts the gut microbiota closer to that of human milk-fed infants and improves intestinal immune defense: a randomized controlled trial
Source: Am J Clin Nutr. 2021 Oct 7;115(1):142–53. doi: 10.1093/ajcn/nqab336 (PMC8755036; doi:10.1093/ajcn/nqab336)
Supplement: nqab336_Supplemental_File [file nqab336_supplemental_file.zip › Suppl methods_MOS efficacy paper REVISED 04Aug2021.docx]

**Term infant formula supplemented with milk-derived oligosaccharides shifts the gut microbiota closer to that of human milk-fed infants and improves intestinal immune defense: A randomized controlled trial**

*Elvira Estorninos et al.*

**Online supplementary material**

**Supplementary Methods**

Table of Contents

[Detection and quantification of pathogenic species by quantitative PCR (qPCR) 2](#_Toc76992547)

[Validation of the qPCR assays 3](#_Toc76992548)

[Execution of the qPCR assays on the samples of present study 5](#_Toc76992549)

[Fecal organic acid analysis by HPLC 6](#_Toc76992550)

[Fecal calcium analysis by ICP-AES 6](#_Toc76992551)

[Markers of intestinal immunity, permeability and inflammation, analysed by ELISA 6](#_Toc76992552)

[Poliovirus (OPV)-specific IgA and IgG 7](#_Toc76992553)

[References 8](#_Toc76992554)

# Detection and quantification of pathogenic species by quantitative polymerase chain reaction (qPCR)

For quantitative analysis of specific bacterial pathogens in fecal samples, qPCR assays as displayed in the **Listing 1** below were applied on isolated DNA from fecal samples. All assays were validated in a fecal matrix background before application (see below).

**Listing 1.** qPCR bacterial targets and method references

| **Bacterial target** | **Gene targets** | **T_annealing_** | **Primer dilution** | **Mastermix** | **Method reference** |
| --- | --- | --- | --- | --- | --- |
| *Clostridioides difficile* | 16S rRNA | 60 ˚C | Fwd: 500 nM  Rev: 500 nM | SYBR Green^a^ | Rinttilä et al. (1) |
| *Clostridioides difficile* | Commercially available kit; *toxB* gene | 60 ˚C | As described in kit specs | Probe^b^ | Primerdesign Ltd ^TM^ genesig® Standard Kit |
| *Clostridium perfringens* | 16S rRNA | 60 ˚C | Fwd: 500 nM  Rev: 500 nM | SYBR Green | Wise et al. (2) |
| *Campylobacter coli* | Commercially available kit; *cadF* gene | 60 ˚C | As described in kit specs | Probe | Primerdesign Ltd ^TM^ genesig® Standard Kit |
| *Campylobacter jejuni* | Commercially available kit; *cadF* gene | 60 ˚C | As described in kit specs | Probe | Primerdesign Ltd ^TM^ genesig® Standard Kit |
| Enteropathogenic *Escherichia coli* (EPEC) | *eaeA* gene | 60 ˚C | Fwd: 200 nM  Rev: 200 nM | SYBR Green | Guion et al. (3) |
| Enterotoxigenic *Escherichia coli* (ETEC) | Heat-stable toxin (ST) gene  Heat-labile toxin (LT) genes | 60 ˚C  60 ˚C | Fwd: 250 nM  Rev: 250 nM  Fwd: 150 nM  Rev: 150 nM | SYBR Green  SYBR Green | Guion et al. (3) |
| *Klebsiella pneumoniae* | Commercially available kit; *phoE* gene | 60 ˚C | As described in kit specs | Probe | Primerdesign Ltd ^TM^ genesig® Standard Kit |
| *Salmonella spp.* | Commercially available kit; *invA* gene | 60 ˚C | As described in kit specs | Probe | Primerdesign Ltd ^TM^ genesig® Standard Kit |

a. Polymerase used in all “SYBR Green” master mixes is iQ™ SYBR® Green Supermix, Bio-Rad

b. Polymerase used in all “Probe” master mixes is iTaq™ Universal Probes Supermix, Bio-Rad

## Validation of the qPCR assays

The qPCR assays were adapted from references / commercial kits as indicated in **Listing 1** and were first validated in a fecal matrix background according to the MIQE guidelines (4). The following control samples were implemented for the validation:

- Target gene amplified DNA product (using bacterial genomic DNA);
- Fecal DNA (isolated from an adult fecal sample, diluted 10-fold) spiked with target gene amplified DNA product (positive control) (to determine potential inhibition of the assay);
- Purified water (negative control);
- Non-spiked fecal DNA (diluted 10-fold, negative control);
- Fecal DNA spiked with chromosomal DNA from *Campylobacter coli* DSM4689, *Campylobacter jejuni* DSM4688 (ATCC 35560), *Clostridioides difficile* C630derm, *Clostridium perfringens* SM101, *Klebsiella pneumoniae* DSM30104 (ATCC 13883), *Escherichia coli* ATCC43887 (EPEC), *Escherichia coli* H10407 (ETEC) and *Salmonella enterica* DSM17058 (LT2) (specificity testing).
- Fecal DNA containing unknown amounts of target species (positive or negative controls), namely:
  - adult DNA with (at least) *K. pneumoniae*
  - adult DNA with (at least) *C. perfringens* and *K. pneumoniae*
  - adult DNA with (at least) EPEC, *C. perfringens*, *C. difficile* and *K. pneumoniae*
  - infant DNA with (at least) *C. jejuni*

The slope, correlation coefficient and PCR efficiency were determined using values of three calibration curves on a single plate. The Cq_min_ and Cq_max_ values (minimal and maximal number of amplification cycles) for each assay were determined using these calibration curves. Accuracy and intermediate precision of the assays were determined using three replicate measurements of three different dilutions (10x, 100x and 1000x) on three replicate plates. Accuracy was calculated as the difference between the experimentally measured value and the true value and is indicated in fold-change differences. The intermediate precision was calculated as a measure of the variation between plates using average and standard deviation values of quadruple measurements in two dilutions (100x and 1000x) of fecal matrix DNA background with spiked target gene amplified product in six concentrations (10^2^ – 10^7^ copies/mL). The limit of detection and quantification (LOD/LOQ) was estimated using the lowest and the highest reliable Cq values of the standard curves of 10 replicate measurements. Any reliable measurement obtained with a value above 1 but below the LOD, was still interpreted to reflect a probable quantification of the bacterial DNA present in the total DNA isolated from the fecal sample. Because the value is a true value (>1) but below the LOD, the value was included in the analyses, but renumbered to ½ LOD. Assay validation results are summarized in the **Listings 2-5**.

**Listing 2** Validation values for *E. coli* qPCR assays in a fecal matrix background. The following criteria were used for acceptance of the validation outcome: slope; -3.1 to -3.8 (perfect slope is -3.3), correlation coefficient (R^2^); ≥0.98 (perfect R^2^ is 1.0), PCR efficiency; 90 – 110% (perfect is 100%), accuracy; <10-fold (perfect is 1.0-fold), precision; <35%.

|  | **EPEC** | **ETEC lt** | **ETEC st** |
| --- | --- | --- | --- |
| Linear dynamic range | 1E8 – 1E1 | 1E8 – 1E1 | 1E8 – 1E1 |
| Slope | -3.371 | -3.450 | 3.312 |
| Correlation coefficient (R^2^) | 0.982 | 0.993 | 0.987 |
| Cq_min_ and Cq_max_ | 9.4 - 33 | 11.1 - 35 | 10 – 32.9 |
| PCR efficiency | 98% | 95% | 100% |
| Accuracy | 1.0 – 1.7 | 2.1 - 2.4 | 1.2 - 1.5 |
| Intermediate precision | 11 – 34% | 10 – 25% | 5 – 33% |
| LOD/LOQ | 10^1^ - 10^2^ copies/µL (=75 - 750 copies/mg) | 10^1^ - 10^2^ copies/µL (=75 - 750 copies/mg) | 10^1^ - 10^2^ copies/µL (=75 - 750 copies/mg) |

No positive signals >LOD were obtained against negative control species for any of the assays, meaning that the qPCR assays are specific for EPEC and ETEC.

**Listing 3** Validation values for *Clostridioides difficile*  and *Clostridium* *perfringens* qPCR assays in a fecal matrix background. The following criteria were used for acceptance of the validation outcome: slope; -3.1 to -3.8 (perfect slope is -3.3), correlation coefficient (R^2^); ≥0.98 (perfect R^2^ is 1.0), PCR efficiency; 90 – 110% (perfect is 100%), accuracy; <10-fold (perfect is 1.0-fold), precision; <35%.

|  | ***C.* *difficile* (16S)** | ***C. difficile* (*toxB*)** | ***C. perfringens*** |
| --- | --- | --- | --- |
| Linear dynamic range | 1E7 – 1E1 | 1E6 – 1E1 | 1E8 – 1E1 |
| Slope | -3.316 | -3.677 | 3.430 |
| Correlation coefficient (R^2^) | 0.967 | 0,997 | 0.992 |
| Cq_min_ and Cq_max_ | 8.8 - 28 | 15.4 – 33.4 | 4.5 – 29 |
| PCR efficiency | 100.3% | 87% | 95.6% |
| Accuracy | 1.0 – 2.0 | 0 – 4.6 | 1.0 – 4.2 |
| Intermediate precision | 8 – 37% | 3 – 28% | 3 – 38% |
| LOD/LOQ | 10^1^ - 10^2^ copies/µL  (=7.5 - 75 copies/mg) | 10^1^ - 10^2^ copies/µL  (=75 - 750 copies/mg) | 10^1^ - 10^2^ copies/µL  (=1.25 – 12.5 copies/mg) |

No positive signals >LOD were obtained against negative control species for any of the assays, meaning that the qPCR assays are specific for the *Clostridium* species they are meant to target.

**Listing 4**. Validation values for *Campylobacter* spp. qPCR assays in a fecal matrix background. The following criteria were used for acceptance of the validation outcome: slope; -3.1 to -3.8 (perfect slope is -3.3), correlation coefficient (R^2^); ≥0.98 (perfect R^2^ is 1.0), PCR efficiency; 90 – 110% (perfect is 100%), accuracy; <10-fold (perfect is 1.0-fold), precision; <35%.

|  | ***C. jejuni*** | ***C. coli*** |
| --- | --- | --- |
| Linear dynamic range | 1E6 – 1E1 | 1E6 – 1E1 |
| Slope | -3.77 | -3.537 |
| Correlation coefficient (R^2^) | 0.996 | 0,993 |
| Cq_min_ and Cq_max_ | 16.1 – 34.7 | 16.8 – 34.2 |
| PCR efficiency | 84.4% | 92.4% |
| Accuracy | 1.0 – 4.8 | 1.0 – 1.9 |
| Intermediate precision | 22 – 33% | 15 – 36% |
| LOD/LOQ | 10^1^ - 10^2^ copies/µL  (=75 - 750 copies/mg) | 10^1^ - 10^2^ copies/µL  (=75 - 750 copies/mg) |

No positive signals >LOD were obtained against negative control species for any of the assays, meaning that the qPCR assays are specific for the *Campylobacter* species they are meant to target.

**Listing 5.** Validation values for *Klebsiella pneumoniae* and *Salmonella* spp. qPCR assays in a fecal matrix background. The following criteria were used for acceptance of the validation outcome: slope; -3.1 to -3.8 (perfect slope is -3.3), correlation coefficient (R^2^); ≥0.98 (perfect R^2^ is 1.0), PCR efficiency; 90 – 110% (perfect is 100%), accuracy; <10-fold (perfect is 1.0-fold), precision; <35%.

|  | ***K. pneumoniae*** | ***Salmonella* spp.** |
| --- | --- | --- |
| Linear dynamic range | 1E6 – 1E1 | 1E6 – 1E1 |
| Slope | -3.731 | -3.862 |
| Correlation coefficient (R^2^) | 0.993 | 0,993 |
| Cq_min_ and Cq_max_ | 16.2 – 34.6 | 16.5 – 34.6 |
| PCR efficiency | 85.3% | 81.5% |
| Accuracy | 1.0 – 8.8 | 1.4 – 6.9 |
| Intermediate precision | 8 – 41% | 19 – 33% |
| LOD/LOQ | 10^1^ - 10^2^ copies/µL  (=75 - 750 copies/mg) | 10^1^ - 10^2^ copies/µL  (=75 - 750 copies/mg) |

No positive signals >LOD were obtained against negative control species for any of the assays, meaning that the qPCR assays are specific for *Klebsiella pneumoniae* and *Salmonella* spp.

## Execution of the qPCR assays on the samples of present study

Validated qPCR assays were applied on the infant fecal DNA samples in three dilutions (10x, 100x and 1000x) using conditions stated in **Listing 1**, in single measurements. Values were deemed reliable when within the Cq_min_ and Cq_max_ values of the assay (**Listings 2-5**)) and within the ΔCq limits (2.2 – 4.6 for each assay). The total number of copies per µl was calculated using the standard curve of the assay and used for calculation of the total number of copies of target specific DNA present in the total microbial DNA isolated from the fecal samples using the following formula: (copies per µl/2)*200/250 = copies per mg.

# Fecal organic acid analysis by HPLC

For organic acid analysis, 250 mg of homogenized fecal sample was diluted with 1 ml of 1M perchloric acid (HClO_4_) to release the organic acids. Lipids and proteins in the fecal sample were removed by centrifugation for 5 min at 20,000 g. Organic acids lactate, acetate, propionate, butyrate, isobutyrate, isovalerate and valerate were determined by high‐performance anion‐exchange chromatography with UV and refractive index detection. 25 µl of the supernatant was injected on a guard column in series with 2 Rezex ROA‐Organic Acid H+ Analytical Columns (Phenomenex, Torrance, CA, USA). The organic acids were eluted isocratic with 5 mM sulfuric acid (H_2_SO_4_) with a flow rate of 0.60 ml/min. The column oven was held at a temperature of 60°C. Data analysis was performed with Chromeleon software v.7.2 (Thermo Fisher Scientific). The result was calculated using two concentrations of a standard mixture containing all relevant organic acids, which was used as a reference sample in each continuous series of analysis (5).

# Fecal calcium analysis by ICP-AES

To measure fecal calcium concentration, 1 ml of 5% trichloric acid (TCA) was added to 150 mg of fecal sample. The sample was incubated at RT for 1 hour and subsequently centrifuged (10 minutes at 14,000 g). The supernatants were diluted with 0.5 g/L CsCl and analyzed by Inductively Coupled Plasma Atomic Emission Spectroscopy (ICP-AES; Varian)[2]. The calcium content of each stool sample was then calculated after accounting for the dilution factor and added water (6).

# Markers of intestinal immunity, permeability and inflammation, analysed by ELISA

Fecal extracts for ELISAs of Alpha 1-Antitrypsin, Beta defensin-2, Calprotectin, Elastase, Lipocalin, Neopterin and total sIgA were prepared using the IDK Extract® extraction buffer (Immundiagnostik AG, Stubenwald-Allee 8a, D-64625 Bensheim). This resulted in an initial dilution of the fecal samples of 100x (100 mg feces in 10 mL extraction buffer). For ELISA of Myeloperoxidase, the “Washing buffer” (Immundiagnostik AG, Stubenwald-Allee 8a, D-64625 Bensheim) was used. Extraction in this buffer resulted in an initial dilution of the fecal samples of 50x (100 mg feces in 5 mL extraction buffer). **Listing 6** summarizes the ELISA targets, commercial kits used, and indicates the two final dilutions used and the lower limit of detection (LLD) for each assay.

**Listing 6.** Commercial ELISA kits, final dilutions of fecal extracts and LODs

| **ELISA target** | **Commercially available kit** | **Catalogue number** | **Final dilution of feces (including the 100x diluted fecal extract)** | **LLD (µg/g wet weight feces)** |
| --- | --- | --- | --- | --- |
| Alpha 1-Antitrypsin | Immundiagnostik AG, Bensheim, Germany | K6750 | 20,000x  50,000x | 0.33 |
| Beta defensin-2 | Immundiagnostik AG, Bensheim, Germany | K6500 | 200x  500x | 0.020 |
| Calprotectin | Immundiagnostik AG, Bensheim, Germany | K6927 | 2,500x  10,000x | 1.3 |
| Elastase | Immundiagnostik AG, Bensheim, Germany | K6915 | 50,000x  100,000x | 0.40 |
| Lipocalin | Eagle Biosciences, Inc. Nashua, NH | NGL35-K01 | 1,800x  3,600x | 0.040 |
| Myeloperoxidase | Immundiagnostik AG, Bensheim, Germany | K6630 | 500x  2,000x | 0.18 |
| Neopterin | IBL, Hamburg, Germany | RE59321 | 100x  200x | 0.034 |
| sIgA | Immundiagnostik AG, Bensheim, Germany | K8870 | 12,500x  50,000x | 2.22 |

# Poliovirus (OPV)-specific IgA and IgG

ELISAs for poliovirus vaccine (OPV)-specific IgA and IgG were performed by BIFINOVE SAS; 99 rue du Jardin des Plantes; 59000 Lille; France as previously published (7). In detail, 100 mg (for IgA) or 200 mg (for IgG) of freeze dried stool was resuspended in 1 ml (IgA) or 2 ml (IgG) of “Stool Reconstitution Buffer” by vortexing. The suspension was incubated at 4°C for 30 min. Subsequently the sample was centrifuged at 19,000 g for 30 min at 4°C. The supernatant was centrifuged at 25,000g for 30min at 4°C. Two-fold dilution series of supernatant were prepared with “Dilution Buffer” supplemented with 10% (V/V) protease inhibitor in a sterile tube. OPV antigen was diluted 1:100 in “Coating Buffer”. The wells of the PVC microtiter plate were coated by adding 100 μl of the OPV dilution and overnight incubation at 4°C while sealed. Next, the coating solution was removed and the plate was washed three times with “Washing Buffer” using an automated microplate washer. The remaining protein binding sites in the coated wells were blocked by adding 300 μl “Blocking Buffer” per well and incubation for 1 h at 37°C, while sealed. Then, the blocking solution was removed and the plate was washed three times with “Washing Buffer”. Subsequently, 100 μl of the fecal supernatant was added and the plate was incubated overnight at 37°C, while sealed. Next, the solution was removed and the plate was washed three times with “Washing Buffer”. 100 μl of biotinylated Anti-Human IgA, (diluted 1:2500) in “Dilution Buffer”, was added per well and the plate was incubated for 90 min at 37°C, while sealed. Then the solution was removed and the wells were washed three times with “Washing Buffer”. Next, 100 μl of Extravidin® peroxidase (dilution 1:1000) in dilution buffer, was added per well and the plate was incubated for 90 min at 37°C, while sealed. Subsequently, the solution was removed and the plate was washed six times with “Washing Buffer”. 100 μl of Substrate Solution was added per well and the plate was incubated for 1 h in the dark at room temperature, while sealed. After sufficient color development, 50 μl of the Stop Solution was added per well. The absorbance (optical density) of each well was measured with a plate reader at 492nm and OPV specific antibody concentration was calculated using a calibration line.

# References

1. Rinttila T, Kassinen A, Malinen E, Krogius L, Palva A. Development of an extensive set of 16S rDNA-targeted primers for quantification of pathogenic and indigenous bacteria in faecal samples by real-time PCR. J Appl Microbiol 2004;97(6):1166-77.

2. Wise MG, Siragusa GR. Quantitative detection of Clostridium perfringens in the broiler fowl gastrointestinal tract by real-time PCR. Appl Environ Microbiol 2005;71(7):3911-6.

3. Guion CE, Ochoa TJ, Walker CM, Barletta F, Cleary TG. Detection of diarrheagenic Escherichia coli by use of melting-curve analysis and real-time multiplex PCR. J Clin Microbiol 2008;46(5):1752-7.

4. Bustin SA, Benes V, Garson JA, Hellemans J, Huggett J, Kubista M, Mueller R, Nolan T, Pfaffl MW, Shipley GL, et al. The MIQE guidelines: minimum information for publication of quantitative real-time PCR experiments. Clin Chem 2009;55(4):611-22.

5. Gommers LMM, Ederveen THA, van der Wijst J, Overmars-Bos C, Kortman GAM, Boekhorst J, Bindels RJM, de Baaij JHF, Hoenderop JGJ. Low gut microbiota diversity and dietary magnesium intake are associated with the development of PPI-induced hypomagnesemia. Faseb J 2019;33(10):11235-46.

6. Ten Bruggencate SJ, Snel J, Schoterman MH, Ertmann E, van der Meulen E, Schonewille A, Bovee-Oudenhoven IM. Efficacy of various dietary calcium salts to improve intestinal resistance to Salmonella infection in rats. Br J Nutr 2011;105(4):489-95.

7. Mullie C, Yazourh A, Thibault H, Odou MF, Singer E, Kalach N, Kremp O, Romond MB. Increased poliovirus-specific intestinal antibody response coincides with promotion of Bifidobacterium longum-infantis and Bifidobacterium breve in infants: a randomized, double-blind, placebo-controlled trial. Pediatr Res 2004;56(5):791-5.
