# Supplementary material for: Term infant formula supplemented with milk-derived oligosaccharides shifts the gut microbiota closer to that of human milk-fed infants and improves intestinal immune defense: a randomized controlled trial
Source: Am J Clin Nutr. 2021 Oct 7;115(1):142–53. doi: 10.1093/ajcn/nqab336 (PMC8755036; doi:10.1093/ajcn/nqab336)
Supplement: nqab336_Supplemental_File [file nqab336_supplemental_file.zip › Suppl tables_MOS efficacy paper REVISED 04Aug2021.docx]

**Term infant formula supplemented with milk-derived oligosaccharides shifts the gut microbiota closer to that of human milk-fed infants and improves intestinal immune defense: A randomized controlled trial**

*Elvira Estorninos et al.*

**Online supplementary material**

**Supplementary tables**

**Table of content**

[Supplementary table 1: Mean and median relative abundance of different Bifidobacterium species at 4 months of age in the three feeding groups. 2](#_Toc78548129)

[Supplementary table 2: Mean and median abundance of specific taxa of interest at 4 months of age in the three feeding groups. 3](#_Toc78548130)

[Supplementary table 3: Detailed statistical outcomes of the comparison of the relative abundance of genus Bifidobacterium at baseline, 2.5 and 4 months of age in the three feeding groups stratified by delivery mode. 4](#_Toc78548131)

[Supplementary Table 4. Comparison of the feeding groups for the presence of selected opportunistic bacterial pathogens analyzed by quantitative PCR (pooled analysis for 2.5 and 4 months of age). 5](#_Toc78548132)

Supplementary table 1: Mean and median relative abundance of different Bifidobacterium species at 4 months of age in the three feeding groups.

|  | | | | | | | | | **Dunn's posthoc test** | | |
| --- | --- | --- | --- | --- | --- | --- | --- | --- | --- | --- | --- |
| **Bifidobacterium species** | **Kruskal-Wallis**  **p-value** | **p-value (FDR)** | **CG_**  **mean** | **CG_**  **median** | **EG_**  **mean** | **EG_**  **median** | **HFI_**  **mean** | **HFI_**  **median** | **HFI-CG** | **HFI-EG** | **CG-EG** |
| *choerinum* | 4.83E-27 | 5.31E-26 | 6.97E-06 | 0.00E+00 | 1.49E-05 | 0.00E+00 | 1.21E-04 | 8.00E-05 | < 0.001 | < 0.001 | ns |
| *longum-infantis* | 7.56E-19 | 4.16E-18 | 6.40E-02 | 3.50E-04 | 1.43E-01 | 5.29E-04 | 4.08E-01 | 3.00E-01 | < 0.001 | < 0.001 | ns |
| *bifidum* | 5.12E-05 | 1.88E-04 | 5.84E-02 | 1.65E-04 | 5.57E-02 | 2.14E-02 | 7.87E-02 | 2.22E-02 | < 0.001 | <0.05 | ns |
| *dentium* | 9.34E-05 | 2.57E-04 | 2.77E-02 | 5.02E-05 | 1.19E-02 | 3.62E-04 | 1.06E-02 | 8.72E-05 | ns | <0.05 | < 0.001 |
| *longum-longum* | 2.45E-03 | 4.73E-03 | 1.27E-01 | 6.86E-03 | 2.26E-01 | 1.37E-01 | 7.63E-02 | 2.48E-03 | ns | <0.05 | <0.01 |
| *breve* | 2.58E-03 | 4.73E-03 | 9.40E-02 | 5.62E-03 | 2.00E-01 | 6.69E-02 | 1.79E-01 | 1.38E-02 | <0.01 | ns | <0.05 |
| *lactis* | 1.56E-02 | 2.45E-02 | 8.57E-04 | 0.00E+00 | 0.00E+00 | 0.00E+00 | 0.00E+00 | 0.00E+00 | ns | ns | <0.05 |
| *scardovi* | 1.17E-01 | 1.61E-01 | 1.85E-04 | 0.00E+00 | 1.18E-02 | 0.00E+00 | 5.17E-04 | 0.00E+00 | na | na | na |
| *catenulatum* | 2.63E-01 | 3.00E-01 | 6.16E-02 | 8.29E-05 | 5.87E-02 | 1.20E-04 | 2.10E-02 | 7.62E-05 | na | na | na |
| *pseudolongum* | 2.73E-01 | 3.00E-01 | 3.57E-06 | 0.00E+00 | 5.00E-06 | 0.00E+00 | 0.00E+00 | 0.00E+00 | na | na | na |
| *adolescentis* | 4.44E-01 | 4.44E-01 | 1.39E-02 | 1.32E-05 | 1.36E-02 | 2.64E-05 | 7.73E-03 | 2.32E-05 | na | na | na |

Feeding groups were compared by Kruskal-Wallis test and false discovery rate correction for multiple testing, followed by pairwise comparisons with Dunn’s posthoc test. CG, n=112; EG, n=114; HFI, n=70. CG, control group; EG, experimental group; HFI, human milk-fed infants; ns, non-significant.

Supplementary table 2: Mean and median abundance of specific taxa of interest at 4 months of age in the three feeding groups.

|  | | | | | | | | | **Dunn's posthoc test** | | |
| --- | --- | --- | --- | --- | --- | --- | --- | --- | --- | --- | --- |
| **Genera** | **Kruskal-**  **Wallis p-value** | **p-value (FDR)** | **CG_mean** | **CG_median** | **EG_mean** | **EG_median** | **HFI_mean** | **HFI_median** | **HFI-CG** | **HFI-EG** | **CG-EG** |
| Bifidobacterium | 8.18E-27 | 4.09E-26 | 4.54E-01 | 5.24E-01 | 7.29E-01 | 7.69E-01 | 7.90E-01 | 8.44E-01 | <0.001 | <0.01 | <0.001 |
| Peptostreptococcaceae  unspecified | 3.79E-23 | 9.48E-23 | 4.80E-03 | 2.21E-03 | 4.57E-04 | 0.00E+00 | 7.77E-04 | 0.00E+00 | <0.001 | ns | <0.001 |
| Lactobacillus | 1.66E-03 | 2.08E-03 | 1.88E-02 | 3.95E-04 | 2.93E-02 | 8.15E-03 | 1.48E-02 | 3.41E-03 | ns | ns | <0.01 |

Feeding groups were compared by Kruskal-Wallis test and false discovery rate correction for multiple testing, followed by pairwise comparisons with Dunn’s posthoc test. CG, n=112; EG, n=114; HFI, n=70. CG, control group; EG, experimental group; HFI, human milk-fed infants; ns, non-significant.

| **Dunn's Multiple Comparison Test** | **Difference in rank sum** | **Significant** |
| --- | --- | --- |
| CG_Caesarean_baseline vs CG_Vaginal_baseline | -31.78 | No |
| CG_Caesarean_baseline vs EG_Caesarean_baseline | 14.07 | No |
| CG_Caesarean_baseline vs EG_Vaginal_baseline | -47.46 | No |
| CG_Caesarean_baseline vs HFI_Caesarean_baseline | -70.81 | No |
| CG_Caesarean_baseline vs HFI_Vaginal_baseline | -120.3 | <0.001 |
| CG_Vaginal_baseline vs EG_Caesarean_baseline | 45.85 | No |
| CG_Vaginal_baseline vs EG_Vaginal_baseline | -15.68 | No |
| CG_Vaginal_baseline vs HFI_Caesarean_baseline | -39.04 | No |
| CG_Vaginal_baseline vs HFI_Vaginal_baseline | -88.55 | <0.001 |
| EG_Caesarean_baseline vs EG_Vaginal_baseline | -61.53 | No |
| EG_Caesarean_baseline vs HFI_Caesarean_baseline | -84.89 | No |
| EG_Caesarean_baseline vs HFI_Vaginal_baseline | -134.4 | <0.001 |
| EG_Vaginal_baseline vs HFI_Caesarean_baseline | -23.36 | No |
| EG_Vaginal_baseline vs HFI_Vaginal_baseline | -72.87 | <0.001 |
| HFI_Caesarean_baseline vs HFI_Vaginal_baseline | -49.51 | No |
| CG_Caesarean_2.5mo  vs CG_Vaginal_2.5mo | 3.583 | No |
| CG_Caesarean_2.5mo  vs EG_Caesarean_2.5mo | -21.01 | No |
| CG_Caesarean_2.5mo  vs EG_Vaginal_2.5mo | -67.09 | <0.05 |
| CG_Caesarean_2.5mo  vs HFI_Caesarean_2.5mo | -56.49 | No |
| CG_Caesarean_2.5mo  vs HFI_Vaginal_2.5mo | -98.75 | <0.001 |
| CG_Vaginal_2.5mo  vs EG_Caesarean_2.5mo | -24.59 | No |
| CG_Vaginal_2.5mo  vs EG_Vaginal_2.5mo | -70.67 | <0.001 |
| CG_Vaginal_2.5mo  vs HFI_Caesarean_2.5mo | -60.08 | <0.05 |
| CG_Vaginal_2.5mo  vs HFI_Vaginal_2.5mo | -102.3 | <0.001 |
| EG_Caesarean_2.5mo  vs EG_Vaginal_2.5mo | -46.08 | No |
| EG_Caesarean_2.5mo  vs HFI_Caesarean_2.5mo | -35.49 | No |
| EG_Caesarean_2.5mo  vs HFI_Vaginal_2.5mo | -77.74 | <0.01 |
| EG_Vaginal_2.5mo  vs HFI_Caesarean_2.5mo | 10.59 | No |
| EG_Vaginal_2.5mo  vs HFI_Vaginal_2.5mo | -31.66 | No |
| HFI_Caesarean_2.5mo  vs HFI_Vaginal_2.5mo | -42.25 | No |
| CG_Vaginal_4mo  vs EG_Caesarean_4mo | -99.18 | <0.001 |
| CG_Vaginal_4mo  vs EG_Vaginal_4mo | -91.58 | <0.001 |
| CG_Vaginal_4mo  vs HFI_Caesarean_4mo | -100.6 | <0.01 |
| CG_Vaginal_4mo  vs HFI_Vaginal_4mo | -138.8 | <0.001 |
| CG_Vaginal_4mo  vs CG_Caesaerean_4mo | 3.265 | No |
| EG_Caesarean_4mo  vs EG_Vaginal_4mo | 7.600 | No |
| EG_Caesarean_4mo  vs HFI_Caesarean_4mo | -1.462 | No |
| EG_Caesarean_4mo  vs HFI_Vaginal_4mo | -39.60 | No |
| EG_Caesarean_4mo  vs CG_Caesaerean_4mo | 102.5 | <0.01 |
| EG_Vaginal_4mo  vs HFI_Caesarean_4mo | -9.062 | No |
| EG_Vaginal_4mo  vs HFI_Vaginal_4mo | -47.20 | <0.01 |
| EG_Vaginal_4mo  vs CG_Caesaerean_4mo | 94.85 | <0.001 |
| HFI_Caesarean_4mo  vs HFI_Vaginal_4mo | -38.13 | No |
| HFI_Caesarean_4mo  vs CG_Caesaerean_4mo | 103.9 | <0.01 |
| HFI_Vaginal_4mo  vs CG_Caesaerean_4mo | 142.0 | <0.001 |

Supplementary table 3: Detailed statistical outcomes of the comparison of the relative abundance of genus Bifidobacterium at baseline, 2.5 and 4 months of age in the three feeding groups stratified by delivery mode.

Feeding groups were compared by Kruskal-Wallis test and false discovery rate correction for multiple testing, followed by pairwise comparisons with Dunn’s posthoc test. CG, n=112; EG, n=114; HFI, n=70 except at 2.5 months of age where CG and EG, n=75; HFI, n=72. CG, control group; EG, experimental group; HFI, human milk-fed infants.

Supplementary Table 4. Comparison of the feeding groups for the presence of selected opportunistic bacterial pathogens analyzed by quantitative PCR (pooled analysis for 2.5 and 4 months of age).

| **Bacteria species/target** | **Adjusted odds ratio^1^** | | |
| --- | --- | --- | --- |
|  | EG/CG | EG/HFI | CG/HFI |
| *Clostridioides difficile 16S* | 0.63 (0.50-0.80)^a^ | 0.89 (0.65-1.21) | 1.40 (1.11-1.77)^b^ |
| *Clostridioides difficile toxB* | 0.30 (0.17-0.52)^a^ | 1.39 (0.57-3.37) | 4.62 (1.11-1.77)^a^ |
| *Clostridium perfringens* | 0.94 (0.89-1.00)^c^ | 1.04 (0.95-1.14) | 1.10 (1.02-1.19)^c^ |
| *Klebsiella pneumonia* | 0.96 (0.92-0.99)^c^ | 1.10 (1.01-1.20)^c^ | 1.15 (1.06-1.25)^a^ |
| EPEC | 0.81 (0.53-1.22) | 15.78 (3.95-63.00)^a^ | 19.59 (4.93-77.90)^a^ |
| ETEC LT | 0.80 (0.18-3.51) | 0.78 (0.08-7.14) | 0.97 (0.11-8.28) |
| *Salmonella* spp. | 1.62 (0.48-5.51) | 2.25 (0.47-10.69) | 1.38 (0.26-7.27) |

^1^ Adjusted odds ratio with the 95%CI were calculated based on the pooled occurrence at 2.5 and 4 months of age. For ETEC ST*, Campylobacter jejuni,* and *Campylobacter coli,* pooled occurrence was insufficient for statistical analysis. Odds ratio were calculated using type 3 tests. Superscript letters indicate significantly reduced odds (^a^ p<0.001; ^b^ p<0.01; ^c^ p<0.05). CG, n=112; EG, n=114; HFI, n=70. HFI, breastfed infants; CG, control formula group; EG, experimental formula group; EPEC, enteropathogenic *Escherichia coli*; ETEC, enterotoxigenic *Escherichia coli*; LT, heat-labile toxin; ST, heat-stable toxin.
